# Nutritional Properties and Consumer’s Acceptance of Provitamin A-Biofortified *Amahewu* Combined with Bambara (*Vigna Subterranea*) Flour

**DOI:** 10.3390/nu11071476

**Published:** 2019-06-28

**Authors:** Temitope D. Awobusuyi, Muthulisi Siwela

**Affiliations:** Department of Dietetics and Human Nutrition, University of KwaZulu-Natal, Pietermaritzburg 3209, South Africa

**Keywords:** bambara, provitamin A-biofortified maize, amahewu, protein energy malnutrition (PEM), germination, roasting, consumer acceptability, vitamin A deficiency (VAD)

## Abstract

Amahewu is a fermented non-alcoholic cereal grain beverage, popular in Southern Africa. This study evaluates the possibility of producing an acceptable provitamin A (PVA)-biofortified maize amahewu, complemented with bambara flour, to contribute towards the alleviation of protein energy malnutrition (PEM) and vitamin A deficiency (VAD). Germinated, roasted, and raw bambara flours, were added at 30% (*w*/*w*) substitution level, separately, to either white maize or PVA-biofortified maize flour, and processed into amahewu. Wheat bran (5% *w*/*w*) was used as reference inoculum. Amahewu samples were analyzed for nutritional properties and acceptability. The protein and lysine contents of amahewu almost doubled with the inclusion of germinated bambara. Protein digestibility of amahewu samples increased by almost 45% with the inclusion of bambara. PVA-biofortified maize amahewu samples complemented with bambara were extremely liked for their color, aroma, and taste when compared with their white maize counterparts. The principal component analysis explained 96% of the variation and PVA-biofortified maize amahewu samples were differentiated from white maize amahewu samples. The taste of amahewu resulting from roasting and germination of bambara was preferred in PVA-biofortified maize amahewu, compared to white maize amahewu. We conclude that PVA-biofortified maize amahewu, complemented with germinated bambara, has the potential to contribute towards the alleviation of PEM and VAD.

## 1. Introduction

Protein energy malnutrition (PEM) and vitamin A deficiency (VAD) affects 250 million people globally, with 2.8 to 3 million people being clinically deficient [1]. PEM is one of the most prevalent problems in Africa. PEM results in diseases like kwashiorkor and marasmus [2]. VAD is also a common deficiency, and results in poor eyesight in children and adults. The high prevalence of poor diet and infectious diseases brought about by VAD and PEM contribute largely to malnutrition. In developing countries, diet is mainly composed of carbohydrate-abundant foods, such as maize, cassava, potatoes, and cereals. However, problems arise when these foods are consumed without protein supplementation, which is often the case in developing countries. This is due to lack of knowledge, and lack of access to expensive protein sources [2]. 

Many strategies have been set to intensify the production, availability, accessibility, and consumption of foods rich in micronutrients. These include combining biofortified staple crops with protein rich legumes such as bambara, soybean, and cowpea.

Biofortication is a process that improves the nutrient quality of food crops through agronomic practices, cross breeding or modern plant biotechnology. Biofortification is sustainable and can deliver naturally fortified foods to individuals who may not have access to commercially fortified foods which are more readily available in urban areas [3]. Research has been carried out to improve the nutritional quality, especially protein content and quality, of cereal-based foods from legume proteins and other protein sources, such as whey protein, soybean protein, and okra seed protein, which have been used for fortification [4]. 

Legumes are known to be rich in protein, dietary fiber, and are of high nutritive value [5]. Legumes are also high in vitamins and other micronutrients. The composition of legumes is acknowledged to play a major role in the prevention of metabolic diseases, such as diabetes mellitus [6,7,8] and coronary heart disease [8,9]. 

White maize, which is the major constituent of the solids in amahewu, is deficient in essential amino acids, such as lysine and tryptophan [10]. Maize is also deficient in other micronutrients, e.g., vitamin A. Another contributing factor to the nutritional limitation of amahewu is that the frequently-used source of the inoculum, millet, which contains anti-nutritional factors, such as tannins, that interferes with the bioavailability of nutrients, e.g., amino acids, in the body [11]. A combination of these problems contributes to the nutritional inadequacy of amahewu. A study was conducted to improve the provitamin A content of amahewu through substitution of white maize with provitamin A-biofortified maize. It was reported that the β-carotene levels, found in provitamin A-biofortified maize were well preserved in amahewu samples after fermentation [12]. The product was, however, deficient in protein content and quality; hence, there is a need for the improvement of amahewu in that aspect. 

In this study, bambara is the legume of choice, because, although it is underutilized, it has many qualities: It is drought tolerant, easy to cultivate, grows well in poor soils, and contains high amounts of protein and essential amino acid. However, despite the potential for the use of bambara groundnut in the fortification of cereal grain-based foods, it has not been supplemented with amahewu. In addition, the biofortified maize used in this study has been bred for higher levels of provitamin A, which is higher than white maize (that has no provitamin A). Therefore, this study aims to evaluate the potential of bambara flour for protein fortification of amahewu made with provitamin A-biofortified maize.

## 2. Materials and Methods 

### 2.1. Plant Materials

Two maize types were used in this study: (1) Provitamin A-biofortified maize (PVAH-62), containing 5 mg/kg β-carotene; and (2) white maize, which served as the control. The grain supply of bambara groundnut that we used was obtained from the Makhatini farm station, in KwaZulu-Natal province of South Africa. Provitamin A-biofortified maize and white maize grains were cultivated at the Experimental Farm of the University of KwaZulu-Natal (UKZN). Bambara groundnut served as the protein source, which was used to supplement amahewu. 

### 2.2. Methods

#### 2.2.1. Preparation of Food Products 

The following parameters were considered: Solid/Solvent ratio: One part of maize meal in seven parts of water for the production of amahewu and 0.5% (*w*/*w*) wheat bran as inoculum. This method was used based on the previous study done by Awobusuyi et al. (2016) [12]. Different concentrations (10%, 20%, 30% and 50% (*w*/*w*)) of BGN were tested on amahewu. However, for this research 30%, defatted bambara flour was used as it was the most acceptable from the preliminary experiment carried out. The bambara groundnuts were subjected to two traditional processing methods—roasting, and germination.

##### Preparation of the Bambara Groundnut Flours 

The bambara groundnuts (BGN) were partitioned into three batches, which are raw BGN, roasted BGN and germinated BGN 

##### Roasted Bambara Groundnut Flour 

Bambara groundnuts were graded, cleaned and soaked in warm water for 24 h to allow easy de-hulling. The water was drained and the seeds de-hulled manually and allowed to dry in a hot air oven at 45 °C for 24 h. The seeds were then roasted in an oven at 180 °C for 15 min. Then, the seeds were ground into flour and sieved using a 0.4 mm wire mesh screen.

##### Germinated Bambara Groundnut Flour 

Seeds were sorted, cleaned, graded and soaked in cold water for 24 h. The seeds were germinated for 72 h inside a jute bag with occasional watering at 12 h intervals to guide against mold growth. After germination, the seeds were thoroughly washed, drained and dried at 45 °C in an oven. Then the seeds were ground into flour and sieved through a 0.4 mm wire mesh screen.

##### Raw Bambara Groundnut Flour 

To produce raw flour, bambara groundnuts were manually de-hulled and ground into flour. The dried seeds were then milled and sieved through a 0.4 mm wire mesh screen.

#### 2.2.2. Preparation of White and Provitamin a Maize Flour 

Provitamin A maize and white maize were ground and separately subjected to the same conditions in order to obtain a thin gruel of the maize. Samples of amahewu were prepared according to a traditional method described by regular consumers of amahewu in rural KwaZulu-Natal. The method involved adding one part of maize meal to seven parts of water and then boiling at 90 °C with occasional stirring, for 15 min. The resulting porridge was left to cool to approximately 40 °C. To three of each of the maize samples (both provitamin A and white maize samples), pre-treated, defatted bambara flours at 30% substitution were added, as illustrated in Table 1. To one of each maize sample, no defatted bambara flour was added, and this was fermented to act as controls for each. Wheat bran (W) was added at 0.5% concentration to porridges, and these were allowed to ferment at 37 °C.

#### 2.2.3. Nutritional Composition 

The nutritional composition of amahewu was determined using standard methods. Amahewu samples were pasteurized at 63 °C for 30 min to stop the fermentation process, and then allowed to cool before analysis. 

***Protein***

The protein content of amahewu was determined with a LECO Truspec Nitrogen Analyser (LECO Corporation, St Joseph, Michigan, USA) (AOAC official method 990.03) [13]. Both the controls and amahewu samples were measured in duplicate and placed into a combustion chamber at 950 °C with an autoloader. The following equation was used to calculate the percentage of protein:

(1) % crude protein=% N×6.25.

***Fat***

The fat content of amahewu was determined according to the Soxhlet procedure, using a Büchi 810 Soxhlet Fat Extractor (Büchi, Flawil, Switzerland) (AOAC Official Method 920.39C) [14]. Petroleum ether was used for extraction, and the percentage crude fat was calculated using the following equation,

(2) $$fat=\frac{weight\;of\;residue\;\left(g\right)\times 100}{weight\;of\;sample\;\left(g\right)}$$

Note: Weight of residue = original sample mass − mass of fat extract

***Moisture***

The moisture content was measured according to the Association of Official Analytical Chemists International (AOAC) Official Method 934.01 [14]. The samples were dried in a forced air oven set at 95 °C for 72 h and then determined by weight difference after freeze drying.

***Ash***

Ash was analyzed by combusting the samples in a furnace set at 550 °C for 4 h, following the AOAC Official Method 923.03 [15]. The crucibles were accurately weighed, and their mass recorded. Five grams of the sample was weighed, and a 7 mL glycerol/methanol mixture was added to each dish and allowed to wet all the particles. The crucibles were placed on a hot plate, under a fume hood, and the temperature was slowly increased. The matter in the crucibles was ignited and burnt until all the organic material has volatilized. The samples were turned into ash using a muffle furnace at 550 °C for 4 h and then cooled in a desiccator for 1h. The sample was weighed, and the mass of the residue determined and expressed as a percentage of the whole sample. The equation below was used in calculating the percentage of ash in the sample,

(3) $$\%\;ash=\frac{\left(mass\;of\;sample+crucible\;after\;ashing\right)-\left(mass\;of\;pre-dried\;crucible\right)}{\left(mass\;of\;sample+crucible\right)-\left(mass\;of\;pre-dried\;crucible\right)}\;\times 100$$

***Amino Acids***

The amino acid profile of the samples was analyzed by the Waters API Quattro Micro Method, which consists of a column C18, 1.7 μm, 2.1 × 100 mm and a binary solvent manager. Samples (400 mg) were subjected to Waters AccQ Tag Ultra Derivatization kit; 10 μL of the undiluted sample was added to the Waters AccQ Tag kit constituents and placed in a heating block at a temperature of 55 °C for 10 min. An injection volume of 1 µL was used, and gradient separation was performed using Solvents A and B from the Waters Accutag kit.

***Mineral elements***

Mineral content was determined by the AOAC Method 6.1.2 [16], using the Inductively Coupled Plasma (ICP) Spectroscopy. Ground samples of each amahewu were acid-digested by adding 1 mL of 55% (*v*/*v*) HNO_3_. 

***In-vitro protein digestibility***

The in-vitro protein digestibility of amahewu samples was determined according to the method of [17]. Amahewu sample, 0.2 g was weighed; 35 mL of 0.1 M phosphate buffer; pH 2 containing 1.5 mg pepsin/mL was added. Pepsin and the sample mixture were incubated at 37 °C for two hours, with incessant shaking. Digestion was stopped with the addition of 2 mL of 2 M NaOH. The suspension was centrifuged at 4800 rpm at 4 °C for 20 min, and the supernatant was discarded. The residue was eroded with 15 mL of 0.1 M phosphate buffer, pH 7, and centrifuged. Again, the supernatant was discarded, and the residue was filtered using Whatman’s Number 3 filter paper. The filter paper containing the undigested protein residue was consequently folded and placed in a digestion tube and dried for two hours at 80 °C. The resultant sample was then analyzed for protein, using the micro kjeldahl method.

#### 2.2.4. Consumer Acceptability

Consumer acceptability was carried out among regular consumers of amahewu, between the ages of 18 and 45 (*n* = 70) in Durban, KwaZulu-Natal, South Africa. A preliminary session was held to explain to panelists the importance of the study, the evaluation procedure, and how the sensory attributes of amahewu were to be evaluated. This was to ensure the reliability of the data, before sensory evaluation commenced. Individual consumers (panelists) evaluated the products based on the aroma, mouth feel, taste, color and overall acceptability. The amahewu samples were evaluated using a nine-point hedonic rating scale (1 = dislike extremely; 9 = liked extremely). Refrigerated amahewu samples were served in polystyrene cups. They were labelled with three-digit codes and served in a random order which was obtained from tables of random numbers and random permutations of nine, respectively. Each panelist was provided with water to cleanse their palate between sample tasting. Provitamin A biofortified maize was used to prepare the test amahewu samples, while the white maize was used to prepare the reference amahewu samples.

#### 2.2.5. Statistical Analysis

Nutritional composition data were analyzed using the Statistical Package for Social Science (SPSS version 25.0 SPSS Inc., Chicago, IL, USA). Mean acceptability scores were computed. One-way analysis of variance (ANOVA) was done, and the mean separation was by the Fisher least significance difference (LSD) test (*p* < 0.05). The principal component analysis (PCA) determined the similarity and difference in the acceptability of amahewu products.

## 3. Results

### 3.1. Proximate Composition 

The protein content increased substantially in the amahewu samples composited with bambara flours (Table 2). The protein content of amahewu almost doubled with the inclusion of bambara at the 30% level of substitution. Pre-treating bambara by germination and roasting also significantly influenced the protein contents of the resulting amahewu samples. Amahewu composited with germinated bambara flour (AGBF) showed the highest increase in protein for both provitamin A and white maize amahewu samples. Maize type did not have any major influence on protein levels of amahewu. However, as expected, amahewu without bambara had the lowest protein content for both provitamin A and white maize amahewu samples. Carbohydrate was the major nutrient of amahewu samples ranging from 63–83%. The inclusion of bambara slightly decreased the carbohydrate content of amahewu samples. The carbohydrate content did not vary with the type of maize used in the preparation of amahewu. Both provitamin A and white maize amahewu recorded the highest carbohydrates content. The fat and ash contents in amahewu samples were generally low without the addition of bambara. Pre-treating bambara by germinating and roasting had no significant effect on the ash and fat content of the bambara-containing amahewu samples.

### 3.2. Mineral Composition 

The levels of individual mineral elements in the amahewu samples composited with 30% bambara flour are presented in Table 3. Major minerals in amahewu samples were potassium, followed by magnesium. The addition of bambara in the preparation of amahewu increased some minerals, especially the zinc and iron contents. The iron content of composited amahewu (34–24 mg/kg) was slightly higher compared to unfortified Provitamin A-biofortified maize and white maize amahewu, and so was the zinc level. Some differences were observed with different pre-treatments. Amahewu samples containing the germinated bambara had a slightly high level of these micronutrients compared to those of roasted bambara, which suggests that germination and roasting of bambara may be effective in boosting the mineral profile of amahewu. When comparing the two types of maize, amahewu made with provitamin A maize had a slightly higher content of minerals, including iron, zinc, potassium and magnesium.

### 3.3. Amino Acid Content 

The amino acid profile of amahewu samples composited with 30% bambara flour is presented in Table 4. The major amino acids in all amahewu samples were glutamic and aspartic acid, which may include glutamine and asparagine. The addition of bambara significantly influenced the amino acid profile of the resulting amahewu samples. The major effect of adding bambara was noted on lysine, which almost doubled with the addition of bambara. Lysine is an essential amino acid that is generally known to be deficient in cereals. When comparing the lysine content to the WHO requirement for infant, lysine accounted for 60% of the recommended intake for children compared to 50% with no bambara added, which indicates about 10% increase at 30% level of inclusion of bambara. The lysine content of composited amahewu accounted for 100% requirements for adults. The effect of pre-treating bambara on lysine contents was also noted. Amahewu composited with germinated bambara flour recorded the highest increase as germination has been reported to increase the amino acid content of cereals. Further, the types of maize used in the preparation of amahewu had a major effect on the amino acid profile. In general, provitamin A-biofortified maize samples prepared with added bambara flour showed the highest increase when compared to their white maize counterparts. Lysine, an essential amino acid generally known to be deficient in cereals increased considerably. This apparent increase of lysine could be attributed to the addition of bambara flour.

### 3.4. In-Vitro Protein Digestibility

**Figure 1:**
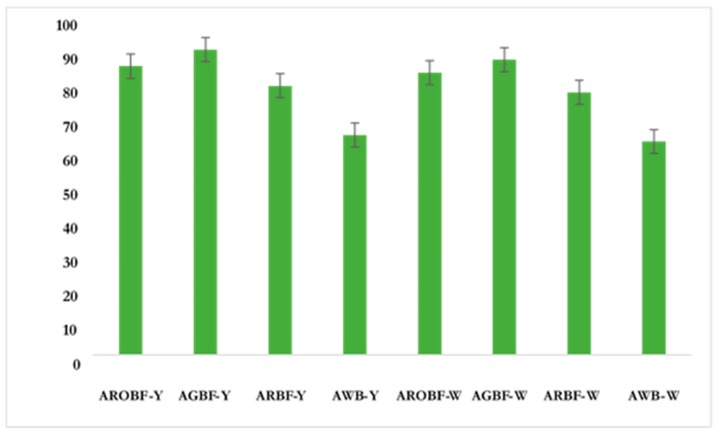
Protein digestibility of amahewu samples.

The in-vitro protein digestibility data are presented in Figure 1. In general, the addition of bambara significantly improved the protein digestibility of amahewu. The protein digestibility of amahewu (65%) increased by almost 45% with the inclusion of bambara. Pre-treating bambara had a major influence on protein digestibility of amahewu. Amahewu composited with germinated bambara flour recorded the highest in vitro protein digestibility increase. The type of maize used does not seem to influence the protein digestibility. Amahewu without bambara flour recorded the least increase in both provitamin A and white maize samples.

### 3.5. Consumer Acceptability

**Figure 2:**
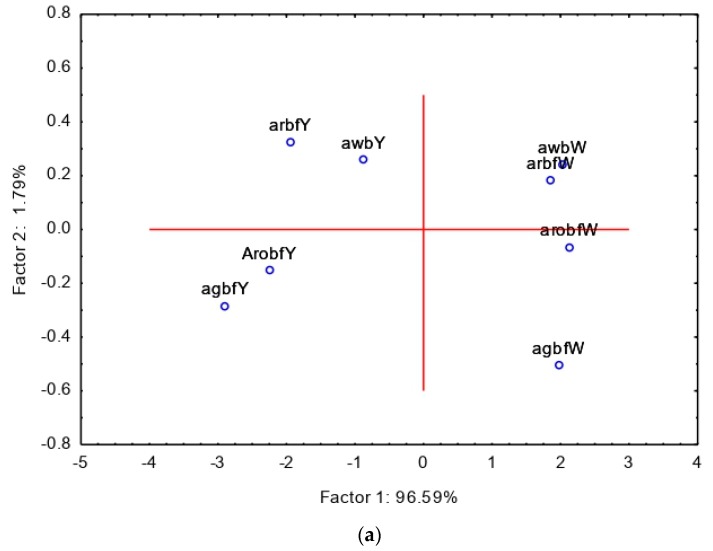

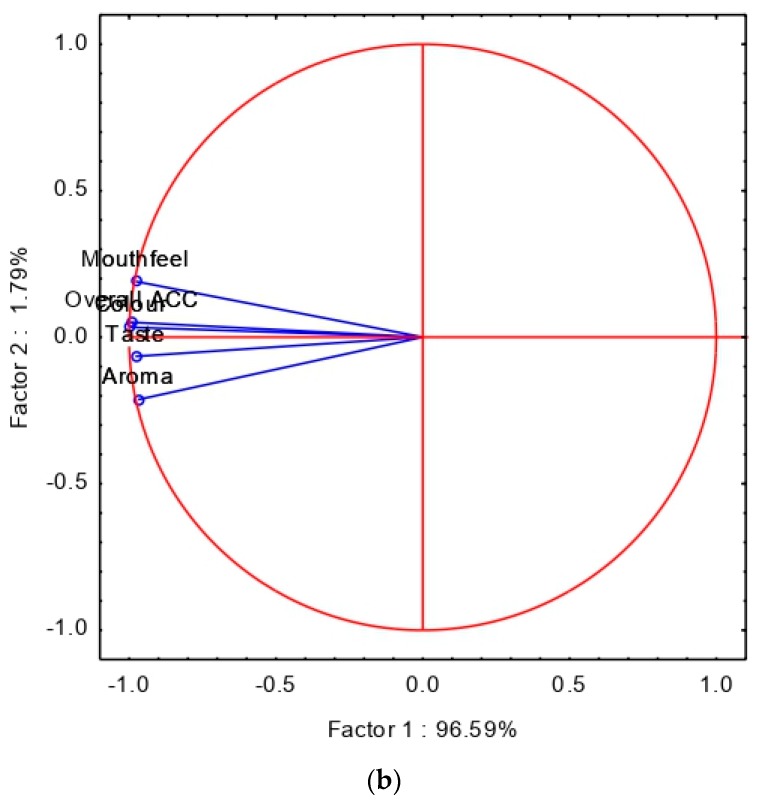
Principal component analysis (PCA) of amahewu samples (Overall ACC means Overall acceptability).

The consumer acceptability data are shown in Table 5. Generally, the addition of bambara did not negatively affect the sensory acceptability of amahewu. The type of maize used in the preparation of amahewu had a major effect on the acceptability of the products. In general, provitamin A-biofortified amahewu samples composited with bambara flour were all/or very much more liked when compared to their white maize counterparts. Provitamin A amahewu composited with germinated bambara flour (AGBF) was the most liked for its color, whilst provitamin A amahewu with roasted bambara flour (AROBF) was the most liked for taste, aroma and overall acceptability. There was no substantial difference in the mouthfeel of the samples, and the products were equally rated (Table 5). Provitamin A and white maize amahewu samples, composited with raw bambara (ARBF), were the least liked for its taste. This could be attributed to the beany flavor associated with bambara. White maize amahewu samples composited with germinated and roasted bambara flour were also more acceptable compared to their counterparts. Amahewu without bambara (control) was the least liked in terms of aroma. Overall, the addition of pre-treated bambara flour to amahewu improved the sensory qualities of amahewu. The principal component analysis (PCA) data are shown in Figure 2a,b. The two components accounted for 98% of the total variation in the sensory attributes data (Figure 2a,b). The first component explained 96% of the variation and provitamin A amahewu samples on the left were differentiated from white maize amahewu samples on the right side of the loading plot. The yellow color and taste of roasted and germinated provitamin A amahewu samples were, somehow, strongly perceived compared to those of white maize amahewu samples. As described above, the taste and color of amahewu combined with provitamin A biofortified maize were extremely liked compared to their white maize counterpart.

## 4. Discussion

### 4.1. Proximate Composition 

The protein content of amahewu combined with bambara flour increased substantially after fermentation. Both provitamin A-biofortified and white maize amahewu composited with germinated bambara (AGBF) showed increased protein. Previous studies reported that germination leads to the synthesis of enzymatic proteins [18]. Proteins contribute to cell growth repair and maintenance, act as enzymes and hormones and maintain a strong immune system by assisting in the increased production of antibodies in response to infections, such as colds, flu, or allergic reactions [19]. Amahewu samples with added bambara flour significantly increased in protein when compared to samples without bambara. Bambara groundnut has been reported to contain a substantial amount (29–30%) of protein [20,21]. These results suggested that bambara could effectively be used in complementation with cereals for improved protein and hence could be applied to combat protein energy malnutrition. 

An increase in protein content was accompanied by a decrease in carbohydrate content of amahewu samples. Microorganisms use carbohydrates as an energy source during fermentation and produce carbon dioxide as a by-product. This causes the nitrogen in the fermented product to be concentrated, and the proportion of protein in the total mass increases [22]. The results show that provitamin A (PVA) maize had higher fat content than white maize, but that did not result in amahewu samples made from PVA maize having a significantly higher fat content than amahewu made from white maize. However, it is worth noting that the higher fat content of the PVA maize is nutritionally significant as fat increases provitamin A and vitamin E bioavailability, as well as bioconversion of beta carotene to vitamin A. The low fat content could have been, due to the oxidation of fat for energy production by inherent organisms. Furthermore, low fat and moisture contents are necessary for the storage quality of the product. A slight increase, although low, was observed for ash.

### 4.2. Mineral Composition

The results obtained showed that the addition of 30% bambara improved the mineral content of amahewu. Provitamin A biofortified amahewu samples recorded the highest increase in minerals among all samples. Amahewu composited with germinated bambara flour (AGBF) had the highest mineral content for both provitamin A and white maize samples when compared to other samples. The zinc and iron contents increased considerably after fermentation. Micronutrient deficiencies affect about two billion persons in the world. Globally, about 740 million people are micronutrient deficient, two billion people are deficient in zinc, and one billion have iron deficiency (anemia) [2]. Zinc is responsible for the storage and release of insulin in the body, and is also responsible for wound healing and other essential body functions [23]. The effect of pre-treatments, such as the roasting and germination process of bambara, could also have contributed to the increase. This result agrees with the work done on fermented maize composited with bambara [24] and fermented porridges, e.g., uji and ugali composited with cowpea [25]. Processing methods, such as germination and fermentation, have been reported to destroy anti-nutritional factors, e.g., phytates, which are responsible for binding the minerals [26]. Heat treatments, such as roasting and frying, have been shown to destroy anti-nutritional factors in legumes [27], this may explain why amahewu composited with roasted bambara flour (AROBF) also recorded a high mineral content after amahewu composited with germinated bambara flour. The minerals become more bioavailable as the level of anti-nutritional factors decreases.

### 4.3. Amino Acid Profile

The results showed a significant improvement in the amino acid profile of amahewu samples with the addition of bambara flour when compared to those without bambara flour. Further, provitamin A biofortified amahewu samples with bambara flour showed the highest improvement in amino acid profile than their white maize counterparts (Table 4). There was a substantial increase in the lysine content of all composited amahewu samples. This is because bambara groundnut is very rich in lysine. Amahewu composited with germinated bambara flour (AGBF) recorded the highest increase for most essential amino acids, including lysine. Earlier studies have documented increased lysine after germination [28]. This agrees with the finding of [29], who showed that germination of cereals and other processing techniques is essential to improve lysine content. Lysine is an essential amino acid, which is vital for growth and maintenance of the body, and are often limiting in some cereals [30,31]. This results showed that 30% bambara groundnut in addition to germination significantly improved the protein quality by elevating the levels of amino acids in amahewu samples and is in agreement with the report of [24,32]. When compared with Food and Agriculture Organization (FAO)/World Health Organization (WHO) standards, the concentrations of all the essential amino acids in all the provitamin A biofortified amahewu samples were generally higher than the pattern of amino acid requirements for adults and accounted for up to 60% for pre-school children.

### 4.4. In Vitro Protein Digestibility

The protein digestibility of amahewu with the addition of bambara groundnut was higher (approx. 93 %), when compared to raw amahewu (amahewu without bambara) (Figure 1). Similar improvement in protein digestibility, following the fermentation of maize gruel, has previously been reported [12,33,34,35,36]. Amahewu composited with germinated and roasted bambara flour had higher in-vitro protein digestibility compared to other amahewu samples. The high IVPD of amahewu composited with germinated bambara flour may be attributed to the effect of germination on anti-nutrient factors. Previous studies have shown that germination could reduce the level of anti-nutritional factors in fermented foods, thus increasing the bioavailability of proteins through the release of proteolytic enzymes and break down of polyphenols [37]. Similar observations have been made on porridges composited with cowpea [25]. Moreover, heat treatments, such as roasting, have also been reported to have an effect of reducing the anti-nutritional factors in legumes, which could have enhanced the IVPD of amahewu [37]. Partial degradation of storage proteins into more simple and soluble products could have also contributed to the increased IVPD [33]. Monawar [35] found that the reduction in pH during fermentation also enhances the activity of native proteolytic enzymes and consequently promotes the breakdown of proteins to smaller polypeptides, which are easily digested by enzymes. Overall, an improvement in digestibility will lead to better protein absorption, and retention in humans following the consumption of protein fortified amahewu with either roasted or germinated bambara flour.

### 4.5. Consumer Acceptability 

The color of provitamin A biofortified maize amahewu samples with added bambara flour was more liked than their white maize counterparts. Provitamin A amahewu composited with roasted bambara (AROBF) and germinated bambara (AGBF) was more acceptable than amahewu samples prepared without bambara (AWB). There was no significant difference between fortified and unfortified amahewu samples made with white maize (Table 5). Previous research found the color acceptability of biofortified maize food products lower than their white maize counterparts [38]. The use of the provitamin A biofortified maize seems to have influenced the color acceptability of the product in a good way. These are very promising findings, because previous research indicates that the unfamiliar color was a major cause of low consumer preference for yellow maize compared to white maize [38,39,40,41,42]. 

The taste-acceptability of amahewu prepared using provitamin A-biofortified maize was higher than that of white maize amahewu (Table 5). However, the taste of provitamin A biofortified amahewu sample with added roasted bambara (AROBF) was more preferred, while amahewu combined with raw bambara (ARBF) was the least liked. Similar observations were made for white maize samples. However, panelists did not find any differences in the mouthfeel of all the samples, and the products were rated similarly. General improvement of product quality after fermentation has been reported widely in the literature by [12,32,43,44]. Amahewu made with provitamin A had higher acceptability scores for aroma compared to their white maize counterparts. However, amahewu combined with roasted bambara was more liked across all samples. Roasting has been reported to produce flavor and aroma compounds. Aman et al. [45] reported that fermentation improves the aroma of fermented maize products, due to the release of aromatic compounds. Overall, provitamin A amahewu samples were more acceptable to consumers than their white maize counterparts.

A principal component analysis (PCA) was used to summarize the variation in the sensory attributes of amahewu samples. Figure 2a shows the projection of scores of amahewu and Figure 2b illustrates loading projections of sensory attributes. The two PCAs described 98% of the total variation in the sensory attributes data. The first principal component (PCA 1) accounted for 96% of the total variation. Provitamin A biofortified amahewu samples were differentiated from their white maize counterparts. PCA indicates that the sensory attributes mainly influencing the overall acceptability of amahewu were color and aroma. It appears that these two sensory attributes largely influenced the overall acceptability of amahewu, because they were intense, and at the same time, highly acceptable, which is the characteristic of fermented foods.

## 5. Conclusions 

The results show that provitamin A-biofortified maize amahewu containing 30% bambara flour was more acceptable as white maize to consumers who regularly consume white maize amahewu.

- One of the objectives of this study was to determine the effect that the addition of bambara groundnut flour will have on the nutritional quality of provitamin A-biofortified amahewu. This study showed that there was an improvement in amino profile, especially an increase in the essential amino acid lysine content, and minerals, such as iron and zinc. This indicates that amahewu containing bambara flour is nutritionally superior to amahewu without bambara. The lysine content of combined bambara/maize amahewu is nutritionally adequate for adults and fairly adequate for age groups lower than five years.
- This study also set out to determine the effect that the addition of bambara groundnut flour will have on the consumer acceptability of provitamin A-biofortified amahewu. The results indicate that consumers prefer provitamin A biofortified maize amahewu over white maize amahewu, and further preferred amahewu with the addition of germinated and roasted bambara over those without any bambara. Roasting of bambara improved the taste, aroma and overall acceptability of amahewu. The consumers used in this study have grown up in a cultural environment where white maize is accepted as traditional food. The findings of this study suggest that there is an opportunity to change the cultural mind-set of preference for white maize.
- The study has demonstrated that the addition of bambara flour with provitamin A biofortified maize, in the form of amahewu, has the potential to contribute to the alleviation of protein and micronutrient (vitamin A, Fe, Zn) malnutrition among the targeted communities, especially the poor rural communities who are highly vulnerable to PEM.

## Figures and Tables

**Table 1:** Partitioning of maize samples.

Provitamin A Maize	White Maize
OBF 30%	OBF 30%
RBF 30%	RBF 30%
GBF 30%	GBF 30%
R0BF 30%	R0BF 30%

OBF = ordinary bambara flour (defatted only); RBF = raw bambara flour (no treatment); GBF = germinated bambara flour; ROBF = roasted bambara flour; wheat bran was added at 5%, based on the percentage solids to serve as an inoculum.

**Table 2:** Proximate composition of amahewu samples (g/100 g, db).

Provitamin-A Products	*CHO	Protein	Fat	Ash	Moisture
AROBF-Y	73 ^c^ ± 0.79	29.7 ^b^ ± 0.25	0.06 ^c^ ± 0.01	0.03 ^a^ ± 0.01	3.16 ^b^ ± 0.03
AGBF-Y	63 ^a^ ± 1.12	34.3 ^d^ ± 0.23	0.08 ^a^ ± 0.01	0.04 ^a^ ± 0.1	3.14 ^a^ ± 0.03
ARBF-Y	69.6 ^b^ ± 0.85	31 ^c^ ± 0.31	0.06 ^b^ ± 0.03	0.03 ^b^ ± 0.05	3.17 ^b^ ± 0.01
AWB-Y	83.3 ^d^ ± 0.32	13.6 ^a^ ± 0.4	0.05 ^b^ ± 0.04	0.02 ^a^ ± 0.01	3.2 ^c^ ± 0.02
**White Maize Products**					
AROBF-W	68 ^b^ ± 0.69	24 ^b^ ± 0.14	0.05 ^c^ ±0.01	0.02 ^a^ ± 0.01	3.16 ^b^ ± 0.03
AGBF-W	63.5 ^a^ ± 1.12	32.3 ^c^ ± 0.23	0.06 ^a^ ± 0.01	0.03 ^a^ ±0.1	3.21 ^c^ ± 0.02
ARBF-W	68.6 ^b^ ± 1.09	28.6 ^b^ ± 0.31	0.06 ^bc^ ± 0.01	0.03 ^b^ ±0.01	3.15 ^a^ ± 0.01
AWB-W	82.8 ^c^ ± 1.45	14 ^a^ ± 0.14	0.05 ^b^ ± 0.04	0.02 ^a^ ±0.01	3.18 ^b^ ± 0.02

*CHO = carbohydrate calculated by difference. Mean with different superscript are significantly different (*p* < 0.05) according to the Fisher least significance difference (LSD) test. AROBF = amahewu + roasted bambara flour; AGBF = amahewu + germinated bambara flour; ARBF = amahewu + raw bambara flour; AWB = amahewu without bambara; Y = yellow provitamin A biofortified maize); W = white maize.

**Table 3:** Mineral composition of amahewu samples (mg/kg, db).

Provitamin A Products					White Maize Products			
Selected Minerals	AROBF-Y	ARBF-Y	AGBF-Y	AWB-Y	AROBF-W	ARBF-W	AGBF-W	AWB-W
Fe	34 ^a^	32 ^a^	38 ^a^	20 ^b^	27 ^b^	24 ^b^	31 ^a^	20 ^b^
Zn	32 ^a^	33 ^a^	36 ^a^	20 ^b^	29 ^a^	23 ^b^	28 ^a^	18 ^b^
K	6960 ^c^	6060 ^c^	8710 ^a^	4055 ^d^	7660 ^b^	6100 ^c^	7720 ^b^	4950 ^d^
Mg	5520 ^b^	4310 ^c^	6640 ^a^	4630 ^c^	4590 ^c^	4300 ^c^	5250 ^b^	4960 ^c^
Na	55 ^d^	80 ^a^	77 ^a^	72 ^b^	59 ^d^	46 ^e^	67 ^c^	74 ^b^
P	210 ^b^	187 ^c^	254 ^a^	182 ^c^	205 ^b^	180 ^c^	212 ^b^	212 ^b^

Mean with different superscript are significantly different (*p* < 0.05) according to the LSD test. Where Y = provitamin A products, W = white maize products.

**Table 4:** Amino acid profile of amahewu samples (g/100 g protein).

Amino Acid	AWB-Y	ARBF-Y	AROBF-Y	AGBF-Y	AWB-W	ARBF-W	AROBF-W	AGBF-W	Preschool Children	Adults
Histidine	1.5	2.1	1.7	2.9	0.8	1.3	1.5	2.2	1.9	1.6
Serine	2.7	3.9	3.0	5.5	1.6	2.3	2.9	3.7		
Arginine	2.5	4.1	2.8	5.0	1.3	2.2	2.7	3.2		
Glycine	1.9	2.8	2.2	3.8	1.2	1.7	2.1	2.8		
Aspartic Acid	3.9	4.9	4.1	7.7	1.9	3.3	4.5	4.5		
Glutamic A	8.9	11	9.9	19	5.8	7.8	10.6	14		
Threonine	1.8	2.6	2.0	3.7	1.1	1.6	1.9	2.7	3.4	0.9
Alanine	3.2	4.4	3.6	6.9	2.4	2.8	3.8	5.7		
Proline	3.6	5.2	4.2	8.4	2.7	3.3	4.2	6.8		
Lysine	2.0	2.6	2.8	3.2	0.8	1.7	1.9	1.9	5.8	1.6
Tyrosine	1.5	2.9	1.8	3.3	1.0	1.4	1.7	2.2		
Valine	2.4	3.3	2.7	4.7	1.4	2.0	2.5	3.4	3.5	1.3
Isoleucine	1.9	2.7	2.1	3.8	1.0	1.5	2.0	2.5	2.8	1.3
Leucine	5.5	8.1	6.3	12	3.9	4.8	6.4	9.2	6.6	1.9
Phenylalanine	2.7	4.2	3.1	5.7	1.5	2.3	3.0	3.6	6.3	1.9

Mean with different superscript are significantly different (*p* < 0.05) according to the LSD test. Food and Agriculture Organization/World Health Organization (1989) recommended pattern (pre-school children aged 2–5 years; adults.

**Table 5:** Consumer acceptability of amahewu samples.

Samples	Colour	Taste	Aroma	Mouthfeel	Overall Acceptability
AROBF-Y	7.7 ^b^ ± 0.6	8.1 ^b^ ± 0.4	8.2 ^b^ ± 0.5	6.8 ^a^ ± 0.5	8.6 ^a,b^ ± 0.6
AGBF-Y	8.1 ^a^ ± 0.7	7.7 ^a^ ± 0.7	8.0 ^a^ ± 0.6	6.9 ^a^ ± 0.6	8.4 ^a^ ± 0.6
ARBF-Y	7.7 ^b^ ± 0.6	7.2 ^b^ ± 0.7	7.5 ^c^ ± 0.5	6.9 ^a^ ± 0.4	8.3 ^b^ ± 0.4
AWB-Y	7.3 ^c^ ± 0.6	7.5 ^c^ ± 0.7	7.0 ^d^ ± 0.5	6.4 ^b^ ± 0.4	8.0 ^c^ ± 0.4
**White Maize Products**					
AROBF-W	6.3 ^d^ ± 0.4	6.3 ^d^ ± 0.4	6.0 ^g^ ± 0.4	6.2 ^d^ ± 0.8	6.9 ^e^ ± 0.5
AGBF-W	6.2 ^d^ ± 0.6	6.1 ^d^ ± 0.5	6.5 ^e^ ± 0.5	6.1 ^d^ ± 0.6	6.7 ^d^ ± 0.4
ARBF-W	6.3 ^d^ ± 0.5	5.8 ^e^ ± 0.6	6.3 ^f^ ± 0.6	6.6 ^c^ ± 0.4	6.6 ^d,e^ ± 0.5
AWB-W	6.2 ^d^ ± 0.4	6.3 ^d^ ± 0.5	5.9 ^g^ ± 0.6	6.4 ^c^ ± 0.5	6.5 ^d,e^ ± 0.8

Mean ± SD (*n* = 70); Mean with different superscript letters are significantly different (*p* < 0.05) according to the LSD test.
